# Combating Antimicrobial Resistance in Singapore: A Qualitative Study Exploring the Policy Context, Challenges, Facilitators, and Proposed Strategies

**DOI:** 10.3390/antibiotics8040201

**Published:** 2019-10-29

**Authors:** Shweta Rajkumar Singh, Alvin Qijia Chua, Sok Teng Tan, Clarence C. Tam, Li Yang Hsu, Helena Legido-Quigley

**Affiliations:** 1 Saw Swee Hock School of Public Health, National University of Singapore, Singapore 117549, Singapore; ephshwe@nus.edu.sg (S.R.S.); alvin.chua@nus.edu.sg (A.Q.C.); soktengtan@u.nus.edu (S.T.T.); clarence.tam@nus.edu.sg (C.C.T.); mdchly@nus.edu.sg (L.Y.H.); 2 London School of Hygiene and Tropical Medicine, London WC1H 9SH, UK

**Keywords:** Antimicrobial resistance, policy analysis, One Health, public health, Singapore

## Abstract

Antimicrobial resistance (AMR) is a global public health threat that warrants urgent attention. However, the multifaceted nature of AMR often complicates the development and implementation of comprehensive policies. In this study, we describe the policy context and explore experts’ perspectives on the challenges, facilitators, and strategies for combating AMR in Singapore. We conducted semi-structured interviews with 21 participants. Interviews were transcribed verbatim and were analyzed thematically, adopting an interpretative approach. Participants reported that the Ministry of Health (MOH) has effectively funded AMR control programs and research in all public hospitals. In addition, a preexisting One Health platform, among MOH, Agri-Food & Veterinary Authority (restructured to form the Singapore Food Agency and the Animal & Veterinary Service under NParks in April 2019), National Environment Agency, and Singapore’s National Water Agency, was perceived to have facilitated the coordination and formulation of Singapore’s AMR strategies. Nonetheless, participants highlighted that the success of AMR strategies is compounded by various challenges such as surveillance in private clinics, resource constraints at community-level health facilities, sub-optimal public awareness, patchy regulation on antimicrobial use in animals, and environmental contamination. This study shows that the process of planning and executing AMR policies is complicated even in a well-resourced country such as Singapore. It has also highlighted the increasing need to address the social, political, cultural, and behavioral aspects influencing AMR. Ultimately, it will be difficult to design policy interventions that cater for the needs of individuals, families, and the community, unless we understand how all these aspects interact and shape the AMR response.

## 1. Introduction

Antimicrobial resistance (AMR) has gained considerable recognition as a major global public health threat [1,2,3,4]. The impact of AMR has been described as a “doomsday scenario” where antibiotics can no longer be relied upon and even minor infections could become untreatable and result in severe morbidity, death and significant economic losses [1,4,5]. The World Health Organization (WHO) released a Global Action Plan for AMR in 2015, setting out five strategic objectives to combat AMR [2]. It also highlighted the importance of a “One Health” approach, requiring collaboration among numerous sectors and actors including human and animal health, agriculture, finance, environment, and well-informed consumers.

Singapore is an island nation with a diverse population of 5.6 million people [6]. Public hospitals cater for 80% of tertiary care services while private sector practitioners account for the majority (80%) of primary care services [7]. There is a large, private animal health sector catering for companion animals, but the agricultural animal health sector is small since Singapore has little local agricultural production [8]. Food requirements are almost entirely supported by imports, which are regulated by food laws focused on ensuring consistent foreign supply of food and agricultural products. Imported food is strictly monitored by the Singapore Food Agency (SFA), while non-food related animal, plant and wildlife management services are provided by National Parks Board (NParks) and Animal & Veterinary Service (AVS) [9]. The National Environment Agency (NEA) and the Public Utilities Board (now known as PUB, Singapore’s National Water Agency) overlooks the public health aspects of environment [10,11].

In line with the Global Action Plan for AMR, Singapore launched its own National Strategic Action Plan on AMR in November 2017 [12]. This multisectoral action plan was jointly developed by the Ministry of Health (MOH), Agri-Food & Veterinary Authority (AVA) of Singapore (restructured to form the SFA and the AVS under NParks in April 2019), NEA, and PUB. It sets a national One Health framework to reduce the emergence and spread of drug-resistant microorganisms through five core strategies: (1) Surveillance and risk assessment; (2) Research; (3) Education; (4) Prevention and control of infection; and (5) Optimization of antimicrobial use. Chua et al. recently published a paper on AMR in Singapore, detailing the changes in efforts against AMR in Singapore from 2008 to 2018 [13]. The paper mentioned that although a better understanding of AMR as a One Health issue has developed, with significant efforts in improving antibiotic prescribing and controlling AMR in Singapore, it was largely focused on the public hospital setting. Even then, a recent national point prevalence survey conducted across 13 acute private and public hospitals in Singapore reported that more than half the patients in acute hospitals were on at least one systemic antibiotic, a much higher proportion compared to inpatients in European hospitals [14]. Based on the few published AMR epidemiology papers, the actual impact on the control of AMR has been mixed, with better control of methicillin-resistant *Staphylococcus aureus* (MRSA), coupled with an increase in resistant Gram-negative bacteria in the hospitals and the community [15,16,17,18]. It has been suggested that more efforts are required in the various sectors, especially the animal/farming and environment sectors, for better AMR control [13].

The AMR issue has been described as a “wicked problem”, where there are many stakeholders involved from the various sectors and several ways to frame the problem [19]. This results in a policy arena where politics and conflict are very evident, and can potentially overwhelm policymakers in devising plans to tackle AMR with a One Health approach. Insights into the policy process for AMR will help to disentangle this complexity and provide a better understanding of support and opposition by key stakeholders for alternative AMR mitigation policies [19]. To date, no qualitative study has been conducted to analyze the factors that can aid in successful implementation of AMR policies in Singapore. Therefore, the aim of our study is to discuss the facilitators and challenges to addressing AMR and the dynamics in the policy process for AMR in Singapore via in-depth interviews. In this study, we interviewed experts in human, animal, and environmental health from both public and private sectors to gain their insights on the policy context, AMR awareness, its emergence in Singapore, and steps taken to mitigate AMR generation and spread. We subsequently identified facilitators and key challenges, and discussed proposed strategies for creating more awareness and combating AMR.

## 2. Materials and Methods

### 2.1. Data Collection

This qualitative study was conducted from September 2017 to May 2018. We used a purposive sampling method to recruit informants in key roles across sectors relevant to the issue of AMR in Singapore. Potential participants were identified from experts in the area of AMR in Singapore, and were approached for an in-depth interview via email explaining the purpose of research. A total of 21 participants from within and outside Singapore were recruited in this study (Table 1). The participants held expertise in human, animal, or environmental health and worked at either ministry, healthcare institution, academia, or civil society. Most participants were from the human health sector. We had difficulty recruiting participants from the environmental sector as potential participants showed more reservation in accepting the invitation to be interviewed, perhaps based on less knowledge about AMR. We were eventually able to recruit an academic and public health official from the environmental sector.

Face-to-face in-depth interviews were conducted by three of the researchers (S.R.S., S.T.T. and H.L.-Q.) in English. Among the interviewers, two were Research Associates and one was an Associate Professor. All interviewers were trained in qualitative research. The interviews were conducted in a quiet space at the preferred location of the participants. Each interview lasted an hour on average and was audio-recorded. There were no repeat interviews. A semi-structured question guide was used to explore the main areas of concern for the participants, in their fields and areas of interest. The question guide was developed with questions focusing on participants’ experiences and perceptions of AMR policy and practice including awareness, key challenges and facilitators in their particular area of expertise, and possible strategies and solutions to address key challenges in the Singaporean context. Participants were not remunerated for the interview.

### 2.2. Ethics

Ethical approval was obtained from the National University of Singapore Institutional Review Board (NUS-IRB). Each participant was provided an information sheet stating the objectives and methods of the research, at the point of recruitment. The confidentiality and anonymity of participants’ responses was also highlighted. Written consent was sought before the beginning of each interview, requesting for permission to be audio-recorded and to be quoted anonymously in research outputs. Participants could refuse any of these options, as well as any questions posed to them during the interview itself. All participants were de-identified to maintain confidentiality.

### 2.3. Data Analysis

All interviews were transcribed verbatim and identifying data were removed from all research documents to ensure confidentiality. QSR NVivo 11 software was used to organize and share the data among study team members. We used an interpretive approach which focuses on the participants’ perceptions and interpretations of the topic of discussion. Thematic analysis was used to inductively identify themes from the data. We drew on techniques from the constant comparative method, such as line by line analysis, naming each line and segment of data, and the use of subsequent interviews to test preliminary assumptions. In addition, deviant cases were explored and examined [20,21]. The conduct of the interviews and coding occurred concurrently to enable the researchers to determine when theme saturation had been reached and to cease recruitment. Thematic saturation was established when the research team discussed and agreed that no new themes were emerging from the data. To improve the credibility of our findings, we conducted a member check at the final stage of manuscript preparation to validate our interpretation of the data and to ensure an accurate representation of participant perspectives. We were unable to contact a few of the participants as they have moved on to another role and their contact information was not readily available. See Supplementary Materials for the Consolidated criteria for Reporting Qualitative research checklist (COREQ form).

## 3. Results

We present our findings under five main themes identified from analysis of participants’ responses. The first theme examines the policy context and discusses whether AMR as a policy issue is a priority in Singapore. The second theme discusses the level of AMR awareness among policymakers, professionals, and the general population. The third and fourth theme discusses the perceived facilitators and challenges to combat AMR in the Singaporean context. Finally, the fifth theme reflects on the reported strategies to increase awareness and combat AMR based on participants’ perceptions.

### 3.1. The Policy Context

Policymakers that participated in the study reflected on the importance of addressing infectious diseases in the Singaporean context, especially after the outbreaks of severe acute respiratory syndrome (SARS) and H1N1 influenza which took place in 2003 and 2009, respectively. As a physician highlighted:

“A lot of incidents drive behavior in Singapore, like for example SARS drove infection control to the front of everything. If you speak to some people, (they) will say infection control became prominent feature with SARS happening.”

—I11, Human Health

In 2009, the MOH initiated the National Antimicrobial Taskforce (NAT) that helped lay down the basics of mandatory surveillance, stewardship, and infection prevention principles in public restructured hospitals. The NAT was tasked to formulate hospital programs and policies, as well as to implement measure for monitoring and evaluation of these policies. It was later reorganized into the National Antimicrobial Resistance Control Committee (NARCC) in 2014. Participants highlighted that the strategy adopted to keep efforts sustainable was by working with people on the ground to reinforce the implementation of these programs. The MOH’s knack for looking at global health issues and comparing itself to other countries also helped in funding and formulation of AMR strategies. The next quote highlights the ways in which the MOH operates including the dedicated funding for antimicrobial stewardship programs:

“The Ministry keeps an eye out for how it compares itself to other countries. So that drives their interest in looking at where we stand, how good, how bad (…) They directly fund quite a lot of the manpower in the hospital systems, certainly for antimicrobial stewardship.”

—I02, Human Health

Participants also remarked that the ministry looked at other countries and international organizations, such as the WHO and the European Union, for inspiration and implemented policies relevant to the Singapore context.

“What WHO or even the EU is recommending, (…) I have been trying to push for that. We follow what others are doing, we don’t want to reinvent the wheel, so that we can then compare ourselves.”

—I07, Human Health

Participants credited the proactive Singaporean government with forming a One Health platform, maintaining effective communication, and coordination between the agencies. One example of this was the formulation of the National Strategic Action Plan on AMR that was released in 2017. Despite establishing a One Health platform, participants suggested that the implementation of programs against AMR would be more challenging. They stressed on the necessity of advocating the importance of AMR to raise awareness.

“Bringing different people to the table in the first instance in Singapore is easy, for somebody that is in charge (…). But at the end, (…) we need to get every single stakeholder to believe in the topic. There’s a lot of sales or advocacy involved.”

—I12, Human Health

### 3.2. AMR Awareness

Most participants recounted low awareness of AMR among Singaporeans. Furthermore, AMR was identified as a recently coined term and many professionals including physicians were not familiar with the term even though they were knowledgeable of the topic itself. The next quote is an example of such reports:

“I guess AMR, this phrase (…) if you were to tell me it’s AMR, I would not know it’s antimicrobial resistance.”

—I01, Human Health

Participants also remarked there was low awareness of AMR both as a topic as well a term among other One Health stakeholders including farmers and others within the environmental health sector. When we contacted potential participants from the environment sector, very few agreed to be interviewed since many reported not having conducted sufficient work related to AMR. The next quote highlights the concerns expressed by an environmental health expert who was worried about the lack of awareness among his colleagues at “the ground level”:

“At the ground level, they were saying, “What is AMR?” People don’t really know. (…) How do we address it? This is a difficult question.”

—I09, Environment Health

Awareness was also perceived to be low in the general population as expressed by participants. Timely awareness efforts by the academic sector had not yet reached the general population. Since 2016, Singapore has been actively participating in the annual World Antibiotic Awareness Week every November, reaching out to the public at various public forums, hospitals and national libraries [22,23]. In 2017, the MOH and Health Promotion Board organized a social campaign focusing on the general prevention of infection to fight the spread of infectious diseases such as influenza and hand, foot and mouth disease [24]. This was closely followed by a “Use Antibiotics Right” campaign with the key message that antibiotics do not work on viruses [25]. Despite these efforts many participants voiced the lack of civil society or social groups movements to support the agenda of AMR in contrast to the high HIV awareness in the early 90s. The following quote highlights the need to further develop health promotion campaigns:

“I think we need people who can inform on public policy in a way that would make our population understand. So we don’t have those communication agencies. I think we have think tanks, right? And we have scientific groups. But we don’t quite have that translation into a public health campaign.”

—I02. Human Health

Some participants stated the importance of raising AMR awareness to counter its emergence. It was suggested that by raising awareness of the topic and getting more funds to drive AMR programs for the general population and stakeholders alike, AMR emergence could be tackled better.

“The community somehow must come to feel that this is an important issue. And once they do, then, you know, naturally, I think all the other pieces will fall into place. The politicians will sing the same song. The private sector will start putting money into the issue.”

—I06, Animal Health

Most stakeholders considered AMR a significant threat to Singapore and global health, but they also mentioned that it was not given the highest priority in the list of local healthcare challenges until several outbreaks of healthcare-associated infection changed the perception of policymakers and administrators with regards to its risk and standing. Until the early 2010s, issues such as ageing population and chronic diseases were rated much higher in priority compared to infectious diseases.

“We had this roundtable discussion. Everybody, all the movers and shakers, public health systems… and they were asked to state these priorities. And believe it or not, infection, the scourge of infection wasn’t one of the top five.”

—I02, Human Health

### 3.3. Challenges to Addressing AMR

#### 3.3.1. Cultural Aspects Influencing AMR

There were many reports suggesting a lack of awareness of AMR from the public. Many participants reiterated the fact that General Practitioners (GPs) may succumb to the pressure of demanding patients to prescribe antibiotics even when they were not clinically indicated. The next quote highlights how patients are “always asking for antibiotics”:

“Patients will always come in and ask for antibiotics. So I think it’s something that we should control and not say that it all is well and then we ignore it. But like I said (…) I think the awareness may not be strong enough for us to continue to pursue.”

—I01, Human Health

It was also highlighted that there is a need for a firm drilling of infection prevention and control practices right in formative years of training for all healthcare professionals. Healthcare professionals needed to be more aware of and prioritize infection control, as it is not currently under “anyone’s radar except for the people who do infection control”:

“As far as infection prevention goes, infection prevention and antimicrobial resistance is like stepchild of everyone. Because it’s not in anyone’s radar except for people who do infection control. So it’s about making it a culture in everyone’s mind, which is not an easy task.”

—I11, Human Health

#### 3.3.2. Inadequate Infection Prevention and Control Measures

Inadequate infrastructure to facilitate optimal infection prevention and control measures was the most commonly cited challenge in addressing AMR. Though infection prevention and control efforts were highly regulated in both the public and private hospital settings, certain contextual factors such as lack of sufficient single room facility were highly challenging to overcome. A few participants mentioned that the design of hospital wards with one room having six beds or eight beds was not conducive to prevent infections:

“It’s not really our fault. You are sitting here scolding us, that we have a lot of MRSA but it’s because, it’s not poor infection control. You are making us take care of patients in overcrowded hospitals. You know, it’s very different from the US or Europe where most of their rooms are two-bedders. If you have a six-bedder, even if I wash my hands, the uncle there who has a maid, and the maid is talking to the other maid and they all share, you know… and it’s practically, it’s all crowded, all these infrastructure issues.”

—I04, Human Health

#### 3.3.3. Lax Stewardship Policies in Private Health Sector

Overall, participants reported that guidelines and antimicrobial stewardship policies were better established in public-sector hospitals and clinics than their private counterparts. Participants attributed this to strict adherence to the guidelines in the public sector, as well as regular audits and monitoring of antibiotic prescriptions by public-sector pharmacists. The following quote is an example of the audits conducted in the public sector:

“If the pharmacy notice that the patient is coming back repeatedly with antibiotics, they will alert the senior doctor to say that they’ve noticed that the doctor’s pattern tends to prescribe a lot of antibiotics. At the same time, we also conduct audits annually on antibiotics usage.”

—I01, Human Health

In contrast, it was reported that private hospitals gave less priority to funding for infection control and stewardship policies than public hospitals. The stewardship element was not strictly monitored in the private hospitals and clinics, where it was reported that the MOH does not have so much leverage. This was raised as a concern by some physicians and policymakers when discussing the extension of AMR policies to the private sector:

“I feel, in the restructured setting, you know, it’s much more, I wouldn’t say easier may not be the word, but maybe less challenging because it’s much more homogenous within that institution and you only have to work in your institution whereas we have to work across all these little and then we are spending a lot of time and cracking ourselves in bits and pieces whereas it can all be done, you know, under one, I think, more harmonization, less bickering, less fighting, and then we can get more funding.”

—I18, Human Health

#### 3.3.4. Defensive Medicine Practices and Expensive Diagnostics

Many participants mentioned that there were practical difficulties in being able to perform adequate tests before dispensing or prescribing antibiotics. Hence physicians and veterinarians tended to practice defensive medicine by prescribing broad-spectrum antibiotics.

“If you are not following the stories in Singapore, it’s not too long ago, you notice that the doctors have been held a lot in, they are being suspended for not diagnosing a certain condition (…) So, if you are going to hide under the umbrella of stewardship, are they going to get bailed out? Is someone going to come and rescue them, you know? Pertinent questions. It’s not that people want to practice defense medicine, but that is somehow being construed or misconstrued from what is happening out there.”

—I18, Human Health

Moreover, veterinarians emphasized that the high diagnostics costs were difficult to justify to pet owners as the next quote exemplifies:

“Textbook always tells us to do so (diagnostics) before you start on any medication. But, in a practical sense, hard to because how do I explain $60 more on top of what they are already paying, for them to get a culture and sensitivity back before we give antibiotics?”

—I17, Animal Health

#### 3.3.5. Disagreements between the Human and Animal Health Sectors

Stakeholders differed on their take as to what contributes more to AMR and ultimately which policy may help in reducing it. Physicians reported being aware of the importance of antimicrobial stewardship policies and infection prevention measures to control AMR, but were unsure if these programs alone could alleviate AMR since they believed that AMR could also be generated by antimicrobial use in animal farming as growth promoters. On the contrary, animal health experts believed the animal food industry did not contribute much to the growing resistance since the volume of antimicrobial used over the lifespan of livestock was very low. As reported by an animal health expert:

“Even though the vets use a huge volume of antibiotic in farming, I think the quantity used per animal over a period of time is way less than what we can get from human clinics. Way, way less.”

—I17, Animal Health

#### 3.3.6. Need to Address the Prevention of AMR outside the Human Health Sector

Many participants also expressed that AMR in Singapore had so far only been addressed in the hospital sector and not much had been done regarding the prevention of AMR in the animal and environment health domains. It was also mentioned that within NAT most of its members and emphasis was still focused on the hospital sector:

“It was still very much hospital-focused. The membership (in the National Antimicrobial Taskforce) still basically comprised representatives of every public-sector hospital, and the chairman and the members were still very much concerned about preventing the spread of AMR organisms in hospitals.”

—I07, Human Health

#### 3.3.7. Contamination of Environment Serving as Breeding Sites for AMR

Environmental health stakeholders expressed the potential of AMR emergence from farms, the community, and the environment as a whole. In general, the perception was that Singapore has not established strategies to deal with environmental contamination. Untreated effluents from hospitals was noted to serve as one of the potential threats in case of a sewage leak.

“The primary source for us (…) is, of course, our hospitals, also hospital effluents. We know that there are very high concentrations of all these last resort antibiotics, especially from the ICUs (Intensive Care Units) and all that, and it gets into the wastewater which then goes into the sewerage system, all the way to the wastewater treatment plant.”

—I14, Environment Health

Also, there was a scope for improving the wastewater treatment standard to treat some antibiotic residues so that they do not harbor AMR.

“Even if you look at the wastewater treatment plants, they’re not doing so well in removing all the pharmaceuticals, all the antibiotics. Some can be removed very well, but some cannot. And so, that means they will be discharged and be effluent.”

—I14, Environment Health

Participants reported that antimicrobial laced water leeching in the environment served as breeding sites for AMR as the next quote emphasizes:

“At the treatment end, there’s no problem because their treatment process is rigorous enough to remove all these bugs and all that…But, of course, I think it’s more from the environmental leakage (of antibiotics).”

—I09, Environment Health

#### 3.3.8. Vested Interests

In addition to the above factors, participants also hinted on the vested interest of different stakeholders. For example, it was mentioned that private practitioners make substantial profits when dispensing antibiotics as it added to their income.

“And by dispensing (antibiotics) they (GPs) are also getting the profits. So, there is already a misaligned incentive.”

—I04, Human Health

Also, some participants reflected on the vested interests of pharmaceutical companies in marketing for higher sales of their antibiotics for their own profits.

“There is a conflict of interest already, because, they (pharmaceuticals) are producing, they are just selling to the farmers (…) in a way they are trying to encourage certain resistance.”

—I17, Animal Health

Animal health stakeholders expressed that the not so strict regulations on the import of newer antibiotics with a broader spectrum of activity served as a leeway for some veterinarians to prescribe them even when not clinically needed. Participants pointed out that the abuse of reserved antibiotics favored acquisition of resistance genes by microorganisms, resulting in loss of antibiotic efficacy.

“There are those not very good ones out in the market that actually goes for the most exotic antibiotics because they distinguish themselves in that sense. Some of them actually use what I call the third-level antibiotic as a first line treatment which I personally don’t agree. Of course, they charge exorbitant amount because it’s ‘special’ antibiotics.”

—I17, Animal Health

In addition, owing to a small farming sector, existing veterinarians had limited expertise in agriculture and aquaculture and hence farmers in Singapore were not mandated to attain prescription for accessing antibiotics independently. Participants alluded that farmers not needing to consult a veterinarian for a prescription for antibiotics served in the farmer’s interest.

“Vets were not familiar to treat fish, so farmers didn’t trust the vets to treat their fish, and vets then became very unwilling to treat farm fish. That’s not changed very much, and there are still issues in Singapore. For example, fish farmers can now buy any antibiotic they want by themselves.”

—I15, Animal Health

### 3.4. Facilitators to Addressing AMR

#### 3.4.1. Leading Role of the MOH and Provision of Appropriate Funding

Singapore’s MOH was unanimously praised by all participants in its proactive approach towards AMR and making way for changes amid very complex issues. It was mentioned that the MOH was transparent and accountable and was operating an efficient public healthcare system in Singapore. Yet, as a drawback some participants argued that having a system that relies too much on decisions made at the highest political level makes the general population and service providers inert.

“That’s why I think in Singapore, unless it comes directly from the Prime Minister’s office, it will never move anything.”

—I11, Human Health

In terms of the resources made available to fund AMR programs, it was reported that MOH had effectively funded AMR control programs and research in all public hospitals.

#### 3.4.2. Trust in the Ability of the Government to Design Policies

Most participants affirmed that the Singaporean population and healthcare professionals bestow trust in government programs and policies. The next quote highlights that whether participants agreed or not with the proposed policy, there was a sense that the government is “trying to do a good thing”:

“When the Singaporean government gets into doing something, they tend to do it very thoroughly. (…) You may not always agree with the strategy, but you have to give them full marks for trying.”

—I04, Human Health

In addition, a preexisting One Health platform among MOH, SFA, AVS, NEA, and PUB was perceived to have facilitated the coordination and formulation of Singapore’s AMR strategies.

#### 3.4.3. Stewardship Programs

Laying the foundation of surveillance in public hospitals and making the data transparent was appreciated by all participants. Participants also shared recent advances in the capacity to implement stewardship and infection prevention and control policies among different Singapore institutions including nursing homes.

“We are looking to reaching out to even nursing homes to provide that kind of bold infection prevention, but also infection control.”

—I03, Human Health

#### 3.4.4. Setting up a Surveillance System

AMR indicators, segregated mainly into process and outcome indicators, were set up in the public hospital surveillance system. Some of the process indicators were antimicrobial use for human and animal health, and infection prevention and control measures such as hand hygiene in hospitals. It was suggested that the Singapore government played a prime role in mandating the standardization, transparency, and sharing of data with the NAT.

“We wanted to measure stuff in Singapore, but really to do that you really need the government involved, (…) otherwise if it’s a voluntary basis, and there are certain biases about data. Whereas if it’s systematic surveillance and it’s mandatory you get a more comprehensive and representative sample.”

—I04, Human Health

Participants mentioned that though the AMR surveillance indicators were defined and well set up in the public hospital domain, surveillance needed to be at a national level, extended to private hospitals, primary care and intermediate/long-term care facilities (GPs, community hospitals and nursing homes), as well as animal and environment sectors.

“The indicators we sort of have been following is still hospital-based indicators. We don’t have country-wide, AMR indicators which internationally people are talking about. For example, we don’t know what is the level of antibiotic consumption at the country level, we don’t know what, for example, when people are talking about “oh, for this bacterial infection, what proportion of this bacterial infection is resistant to what antibiotics” you know? We haven’t gone to measuring that kind of level.”

—I07, Human Health

#### 3.4.5. Highly Motivated Workforce

The motivation of the workforce was attributed to the competition, prestige, and reputation of the workforce. In the hospital setting, metrics and outcomes of antimicrobial stewardship such as average defined daily dose and percentage of appropriate antibiotics prescribed, and length of hospitalization for each department are often reported and shared among the various departments within the hospital. The transparency of data highly motivated the workforce to perform well in these benchmarking exercises. The following quote is an example of such occurrence:

“More important I think is the peer pressure and peer standing. And when it’s made the department target and communicated by the head of the department, no one wants to be the worst department in the stats when they are shown up. So there’s lot of department pride and that’s what drives stuff. Doctors are often competitive, some of the nurses too. And no one wants to be the idiot that lets the team down.”

—I03, Human Health

#### 3.4.6. Research Excellence

Research in Singapore was unanimously considered to be well promoted and funded, albeit it was also rated to be very competitive in terms of securing research funding. There was ongoing AMR research including in the areas of surveillance, prevention of transmission, whole genome sequencing, among others. Of the various arms of AMR research, surveillance was considered to be one of the main pillars in AMR research:

“One of the key reasons in the national action plan is research. And among the research activities there have been sort of highlighted in terms of priority, is better surveillance. You know, how to better measure the different perspectives and areas that we need to, outside the hospitals: primary care, the community and understanding the sort of pathways of antibiotic resistances, what is driving, the drivers of antibiotic resistance locally…”

—I07, Human Health

#### 3.4.7. Close Connections between Researchers and Policymakers

The researcher and academic participants mentioned that the government and ministry were very tightly knit in Singapore. Leadership was very open and attentive to research findings that could be translated into better health practices in Singapore. As the following quote highlights:

“There’s that translation angle number one. There is the One Health angle number two, and then of course number three we also engage with basic science collaborators, to try to understand the basic science mechanisms of this issue.”

—I08, Human Health

#### 3.4.8. Role of Singapore in Regional AMR

As AMR is a global health issue, participants remarked that Singapore alone could do very little in controlling AMR as a whole. Despite all policies Singapore will not be untouched by rising AMR in the region which can be ultimately transmitted via large imports of agricultural products and humans travelling for trade and tourism. However, most participants mentioned that Singapore could play a role by coordinating and sharing expertise with neighboring member states of the Association of Southeast Asian Nations (ASEAN).

Many participants proposed the idea that Singapore can serve as a research hub owing to its unique positioning and research capacity. Sharing innovative findings both on science, clinical practice and exemplary policy implementation strategies could prove helpful to neighboring countries that share some contextual factors with Singapore.

“It is an ideal geographic position as well as possibly a good political one with the recognition by the west that Singapore is a high-income country as well as intellectually advanced. That bridge between the west and low- and middle-income countries especially in the Asia Pacific region could really be bridged by your university and your researchers.”

—I20, Human Health

A few ministerial participants affirmed Singapore’s role of coordinating regional efforts on AMR.

“What Singapore is trying to do is trying to harmonize efforts in the agriculture sector and the livestock sector because these are under different ASEAN stream workgroups. So we also work with FAO to coordinate these.”

—I13, Animal Health

### 3.5. Strategies to Raise AMR Awareness and Combat AMR

#### 3.5.1. Projecting AMR Costs

Participants articulated that highlighting the increasing cost of managing patients infected with resistant organisms and the economic loss from AMR could be a potential way of drawing attention to the topic and getting funds to drive programs. As specific examples, participants illustrated not just the costs of treating resistant infections, but also the costs associated with implementing stricter infection control measures.

Some participants believed that healthcare costs would increase due to AMR. These were attributed to many reasons, one of which was the cost of more effective antibiotics required to treat infections with resistant organisms. Another prominent reason mentioned was the costs towards infection prevention and control in healthcare settings. This was considered a necessary step to reduce the risk of healthcare-associated infections, especially in the face of worsening AMR. The following factors were suggested by one participant to prevent the spread of resistant pathogens and to cater to patients harboring resistant or infectious organisms:

“That is going to jack up costs in all kinds of ways. Number 1, you are going to have to pay for all those gowns, gloves, single rooms, for isolation, etc. Number 2 you need more manpower, because suddenly a nurse is going to be slowed down right? Or a doctor. You have to put on all this gear, you are going to be seeing fewer patients in the same amount of time, just because of … it’s a bother.”

—I04, Human Health

#### 3.5.2. Push Forward AMR Agenda by Fear of no Treatment

Many participants believed that unless AMR’s potential damage to the population and the economy is projected, people are not going to take AMR seriously. Some participants mentioned that only a grave incident such as an AMR outbreak would be able to catch the attention of the general public and extend the prevention efforts of AMR.

#### 3.5.3. Building Communities of Practice

A few participants articulated that rather than forcing a top-down policy approach, AMR could be better tackled by the sharing of best practices through communities of practitioners and creating social movements to encourage and sustain behavior change.

“Practitioners come together to share best practices, through the ID networks, through the hospital networks, through the volunteer networks, you know, build communities of practice until this becomes a culture. And people are learning from each other (…) But whether it gets done is a question of culture, a culture needs strategy every day (…) so you want to do change, and you want to do large scale change and you want to sustain the change you need to create a movement not a directive… I think it’s more important to you to create the social movement around what that is, because if you are going to achieve this style change of behavior you need a more behavioral approach to this problem not a clinical approach.”

—I05, Human Health

#### 3.5.4. Engaging the Community and Public Campaigns on the Drivers of AMR

Participants reflected on the need to develop public campaigns to sensitize people to become aware of and understand the fundamentals of programs and strategies against AMR. These include stewardship, adherence to vaccination guidelines, better infection prevention and control practices in hospitals as well as programs in the community. All these programs needed to take into account social, behavioral, and cultural aspects to accommodate the needs and perceptions of the community.

“There is always a need to build a culture and to build a system for people to do the right thing. And the same applies outside the hospitals. So I think you know the schools have hand washing to teach the next generation.”

—I05, Human Health

#### 3.5.5. Increase Vaccination

Most participants asserted that one of the probable strategies to counter AMR was to increase vaccination rates in children and adult population. However, increasing vaccination uptake will uncover another set of issues including population awareness, behavior changes, funding for regular vaccination, workforce to administer vaccination, and availability of vaccines.

## 4. Discussion

This paper has discussed the challenges and facilitators to addressing AMR in a well-resourced setting such as Singapore. Despite Singapore having an established One Health platform representing all the different stakeholders; appropriate funding to address the response; existence of stewardship and surveillance programs in public hospitals; an active program in research; and a motivated workforce; most participants raised several challenges to address AMR efficiently. These included, among other, vested interests among some stakeholders; disagreements between the human and animal health sectors; low public awareness; the need to address prevention of AMR in the animal and environmental sector; political and cultural aspects influencing AMR; and lax stewardship policies in the private sector.

Therefore, the results of this paper highlight how difficult it can be to address AMR, especially when considering that Singapore has well-functioning stewardship programs, comprehensive hospital surveillance systems, and some of the latest technologies and innovations to address it. The example of AMR in Singapore brings to light the increasing need to address the social, economic, political, cultural, and behavioral aspects influencing AMR rather than just focusing on the technical solutions. Unless we understand how all these aspects operate and drive the response, it will be difficult to design appropriate policy interventions. In the next paragraphs we provide an example of each of these aspects to highlight their importance in the Singaporean setting. We address “the social” by analyzing how AMR has been socially constructed in Singapore; we discuss the influence of “politics” by considering power relations and governance approaches for the successful control of AMR; we address “behavioral aspects” by analyzing vested interests; and we consider “cultural components” by exploring how patients’ cultural beliefs influence antibiotic consumption.

First, it is important to understand how AMR has been socially constructed in Singapore and how power relations operate between the different stakeholders. Wernli et al. distinguished five different frames that map the global policy discourse on AMR [26]. These are: a One Health approach combining in one paradigm human, animal, and environment health; a health security threat giving rise to the global health security agenda; a healthcare policy issue with the dominance of the medical profession; a development issue where it is considered that low-and-middle income countries drive AMR; and an innovation issue with a focus on new diagnostics and antibiotics. Wernli et al. also distinguish a set of actors and policies for each of the frames described. In the Singaporean context, it is interesting to note that several frames coexist with their respective actors leading the selected frame. First, it was reported that there is a One Health platform where all stakeholders meet. Second, there is a strong healthcare policy frame as it was reported that Singapore’s AMR policies were largely driven by a group of infectious disease clinicians. Third, AMR is also seen as an innovation issue where researchers are working to identify novel solutions on all fronts. The results of this study suggest that a deeper understanding is needed of the different power relations between these distinct groups to ensure the successful implementation of policies designed to respond to the threat of resistance. However, from our participants’ responses we can tentatively conclude that AMR is predominantly seen as a biomedical problem within the context of healthcare facilities and within the pursuit of clinicians controlling infectious diseases even when they still participate in the One Health platform. It could also be argued that historically the emphasis on AMR has been biomedical because this is where the health burden was seen and therefore where the expertise developed. This, however, does not mean that other areas are not seen as important.

Second, considering governance approaches for the successful control of AMR requires a focus on both whole of society and sector specific approaches [19]. This was detailed in a previous paper where we proposed a new governance framework to investigate power relations and responses for diverse stakeholders addressing AMR [19]. As Kickbusch mentioned, and further discussed in our paper, recently there has been a diffusion of governance moving from a model dominated by the state to a model co-produced by a wide range of actors [27]. As a result, Kickbusch has identified five types of governance present in whole-of-government approaches. These include: (1) governing among others by mixing regulation and persuasion; (2) by collaborating; (3) by engaging citizens; (4) through independent agencies and expert bodies; (5) and by adaptive policies, resilient structures, and foresight.

In the Singapore setting, many participants highlighted the leading role of the MOH in regulating the health sector, but acknowledged challenges in regulating the private and animal health sector. These challenges present examples where governance structures will need to weigh up the balance between regulation and persuasion as policy approaches. In the Singaporean context, differential policies in the public and private human healthcare sectors, whether by design or practice, are likely to be counterproductive, as patients use both sectors and the occurrence of resistant infections in one sector has implications for the other. On the other hand, the agricultural sector in Singapore is small and the availability of adequate veterinary expertise limited; imposing tight regulations on antibiotic access in the absence of qualified prescribers is therefore challenging, and persuasive strategies to reduce antibiotic use in this sector are likely to be more realistic, at least in the short term. On their collaborative approach towards governance, many participants highlighted the One Health platform as a good way of bringing stakeholders together. The good relationship between researchers and policymakers was also emphasized via the many avenues for researchers to discuss and share their research findings and discuss policy implications with policymakers. However, there were also conflicts reported specifically between human and animal health experts. This was crystalized on their different takes on what contributes more to AMR. Most physicians believed that antimicrobial use in animal farming as growth promoters might be a more significant driver of AMR, as has been explained by studies [28]. In contrast, animal health experts believed the animal food industry did not contribute much to the growing resistance when compared to AMR infection rates in the health sector. Finally, governing AMR through the engagement of citizens and civil society was not reported as a strategy in Singapore. However, this is not unique to Singapore, as it is well recognized that globally there has been a lack of engagement of civil society organizations in addressing AMR, especially when comparing and contrasting to the high HIV involvement in the early 90s.

Third, the AMR policy context represents a policy arena where certain groups engage in “behaviors” that might conflict with the overall goal of controlling AMR. Vested interests are more noticeable than in other health policy areas since different professional groups coexist in the AMR policy arena and there is substantial profit to be made from selling antibiotics [29,30]. In Singapore, vested interests were mentioned among pharmaceutical companies in marketing for higher sales of their antibiotics for their own profits; among private practitioners as they make substantial profits when dispensing antibiotics; and among some veterinarians who prescribed antibiotics even when not clinically needed. Our previous research in other settings (i.e., Pakistan and Cambodia) also suggested several considerations and vested interests around antibiotic use which can hinder the control of AMR in the human and animal health sectors [29,30]. For example, it was reported that among doctors there are monetary incentives to prescribe certain antibiotics as these are often negotiated with pharmaceutical companies. It was also recounted that a large proportion of the income at hospital level and in pharmacies comes from prescribing antibiotics and as a result it might be difficult to introduce stewardship programs to curb their prescription. While these reports were present in Cambodia and Pakistan, they were absent in Singapore. Monetary incentives were not mentioned that often by our participants and were absent at hospital level.

Fourth, many participants reported that “cultural aspects” and perception regarding medication influenced patients’ requests for antibiotics, with patients requesting them, and GPs recognizing feeling the pressure to prescribe them. Other research has found similar findings, with Lam et al. observing that primary healthcare physicians over-prescribed antibiotics in order to satisfy their patients [31]. There were also reports suggesting a lack of awareness of AMR from the public and as a result, patients continued demanding antibiotics even when they were not clinically indicated. This was also reported in a Singaporean study that surveyed patients and found that most patients seeking primary health care in Singapore were misinformed about the role of antibiotics, with poor knowledge being associated with wanting antibiotics [32].

To our knowledge, this is one of the first studies to explore the social, political, behavioral, and cultural aspects influencing AMR in a well-resourced setting. A key strength of this study is that we were able to gather the perspectives of multiple stakeholders in the human, animal, and environmental sector. However, we had difficulty in recruiting participants from the environmental sector, and we were only able to interview two participants. A limitation of this study is that participants could have downplayed some of the challenges and some strategies that are considered key to address AMR could have been missed. For example, the fact that Singapore has a well-functioning system for dispensing antibiotics in the human sector, with antibiotics only available with a physician’s prescription, was not mentioned as often as a facilitator as one might expect. This could be the case because it was already taken for granted as it has been in existence for several years.

Finally, participants in this study were asked to describe the most important strategies to combat AMR and to raise awareness among the population. The most often mentioned responses were: the need to project AMR costs to draw more attention to the topic; pushing forward the AMR agenda by fear of no treatment; creating a community of practice; considering the social aspects of AMR in order to develop prevention efforts against AMR; engaging the community and public campaigns on the drivers of AMR; and increasing vaccination rates in children and adult population. One important step to realizing these strategies would be to have good quality data obtained from standardized surveillance platforms at the national level. This will allow the conduct of outcome research, which can eventually be translated into messages to increase awareness and to push forward the AMR agenda. For example, Naylor et al. used a national mandatory surveillance database in England to quantify the cost and mortality burden of *Escherichia coli* bacteremia, as well as the influence different resistances have on them. Such findings will be useful for understanding the health and economic impact of future trends in resistance, and for prioritization of funding and strategies to tackle the problem [33]. In addition, future research could focus on exploring further how the current Singapore National Strategic Action Plan is being implemented; exploring in more detail the social, political, behavioral and cultural components affecting AMR; and analyzing at greater length what type of AMR awareness campaigns could be developed to reach the community and engage civil society organizations.

## 5. Conclusions

The process of designing and implementing AMR policies is complicated even in a country such as Singapore, which has dedicated funding and has developed a multisectoral approach to address AMR. This paper has highlighted the increasing need to address the social, political, cultural, and behavioral aspects influencing AMR rather than just focusing on the technical solutions. Unless we understand how all these aspects operate and drive the response, it will be difficult to design policy interventions that produce the desired results and that cater for the needs of individuals, families, and the community as a whole.

## Figures and Tables

**Table 1:** Participants’ characteristics according to profession and organization.

Sector	Profession	Count (*n*)	Organization	Count (*n*)
Human				
	Medical Practitioners	4	Government agency	3
	Academics	3	Hospital (public and private)	6
	Managers	6	Primary care	1
Animal				
	Academics	2	University	1
	Laboratory Manager	1	Veterinary Clinic	2
	Private practice	2	Government agency	2
	Government agency manager	1		
Environment				
	Policy maker	1	Government agency	1
	Academic	1	University	1
International				
			WHO, NGOs, etc.	4
	Total	21	Total	21

WHO = World Health Organization; NGOs = Non-governmental organizations.

## Supplementary Materials

The following are available online at https://www.mdpi.com/2079-6382/8/4/201/s1, COREQ (COnsolidated criteria for REporting Qualitative research) Checklist.
